# Measurement of Brain Function of Car Driver Using Functional Near-Infrared Spectroscopy (fNIRS)

**DOI:** 10.1155/2009/164958

**Published:** 2009-06-25

**Authors:** Hitoshi Tsunashima, Kazuki Yanagisawa

**Affiliations:** ^1^Department of Mechanical Engineering, College of Industrial Technology, Nihon University, 1-2-1 Izumi-cho, Narashino-shi, Chiba 275-8575, Japan; ^2^Department of Mechanical Engineering, Graduate school of Nihon University, 1-2-1 Izumi-cho, Narashino-shi, Chiba 275-8575, Japan

## Abstract

The aim of this study is to propose a method for analyzing measured signal obtained from functional Near-Infrared Spectroscopy (fNIRS), which is applicable for neuroimaging studies for car drivers. We developed a signal processing method by multiresolution analysis (MRA) based on discrete wavelet transform. Statistical group analysis using Z-score is conducted after the extraction of task-related signal using MRA. Brain activities of subjects with different level of mental calculation are measured by fNIRS and fMRI. Results of mental calculation with nine subjects by using fNIRS and fMRI showed that the proposed methods were effective for the evaluation of brain activities due to the task. Finally, the proposed method is applied for evaluating brain function of car driver with and without adaptive cruise control (ACC) system for demonstrating the effectiveness of the proposed method. The results showed that frontal lobe was less active when the subject drove with ACC.

## 1. Introduction

In recent years, various driving assistance systems have been developed to ensure safety by reducing driver workloads. Examples include the Adaptive Cruise Control (ACC) system, which maintains a safe distance between the driver's vehicle and the vehicle ahead of it and the lane-keeping assistance system, which keeps the car in a lane through steering support.

However, it is also possible that while driver workload is reduced, the driver's attention is also reduced, resulting in unexpected accidents. Therefore, it is necessary to examine driver workload from the viewpoints of cognitive engineering and human physiology. It is necessary to clarify the relationship between driver workload and brain activity, which includes recognition and judgment. It is then necessary to evaluate the driver's attention and to clarify the relationship between brain activity and driving performance. 

A small number of neuroimaging studies using driving simulator examine brain activity in car driving. In these studies [1, 2], functional magnetic resonance imaging (fMRI) has been used. However, fMRI has many shortcomings in evaluating driving performance, because it requires the subject to lie in a narrow cylinder during evaluation and does not permit movement of the body, particularly the head. This situation makes the driving task unrealistic and unnatural.

Near-Infrared Spectroscopy (NIRS) has gained attention in recent years [3, 4]. This noninvasive technique uses near-infrared light to evaluate an increase or decrease in oxygenated hemoglobin or deoxygenated hemoglobin in the tissue from the body surface. 

NIRS can detect the hemodynamic of the brain in real time while the subject is moving. Therefore, brain activity can be measured in various environments. Recent research has used functional Near-Infrared Spectroscopy (fNIRS) to measure brain activity of train driver [5, 6]. Shimizu et al. used fNIRS to evaluate the mental activity of car driver using a driving simulator [7].

Various arguments have focused on interpretation of signals obtained from fNIRS, and no uniform signal-processing method has yet been established. Averaging and base-line correction are conventional signal-processing methods used for the fNIRS signal. These methods require block design, an experimental technique that involves repeating the same stimuli (tasks) and resting multiple times in order to detect brain activation during a task. However, it has been pointed out that brain activation gradually declines when one subject repeats the same task multiple times [8].

Fourier analysis, which is frequently used for signal analysis, transforms information in the time domain into the frequency domain through the Fourier transform. However, time information is lost in the course of transform. As the fNIRS signal has unsteady nature, time-frequency analysis is suitable for the fNIRS signal.

The wavelet transform is an efficient method of time-frequency analysis [9]. It adapts the window width in time and frequency so that the window width in frequency becomes smaller when the window width in time is large, or the window width in frequency becomes larger when the window width in time is small. Multiresolution analysis (MRA) [10] decomposes the signal into different scales of resolution. The MRA with orthonormal wavelet base facilitates complete decomposition and reconstruction of the signal effectively without losing original information of the signal.

In addition to this, oxygenated hemoglobin and deoxygenated hemoglobin measured in fNIRS are relative value from the beginning of measurement, which is changeable for subject and part of the brain. Thus, simple averaging of fNIRS signal should not be applied for statistical analysis. To solve this problem, we propose Z-scored fNIRS signal for statistical analysis.

The aim of the study is to propose the signal processing method suitable for fNIRS signal which is applicable for neuroimaging studies for car drivers using fNIRS. In this paper, we first describe the principle of measurement of brain activity with fNIRS. Then, we propose the discrete wavelet-based MRA to extract the task-related signal from the original fNIRS recordings. We conducted simultaneous measurement experiments with fNIRS and fMRI using mental calculation tasks to confirm the validity of the proposed method. The Z-scored fNIRS signal is proposed for statistical analysis. We show the possibility of the proposed method for evaluating driver's brain activity in realistic driving environment.

## 2. Principle of fNIRS

Using near-infrared rays, fNIRS noninvasively measures changes in cerebral blood flow. Its principle of measurement, which was developed by Jöbsis [11], is based on measurement of oxygenation of hemoglobin in the cerebral blood flow. 

In uniformly distributed tissue, incident light is attenuated by absorption and scattering. Therefore, the following expression, a modified Lambert-Beer law, was used:

(1) $$\text{Abs}=-\log\;\left(\frac{I_{\text{out}}}{I_{\text{in}}}\right)=\varepsilon dC+S.$$

Here, *I*_in_ is the irradiated quantity of light; *I*_out_ is the detected quantity of light; *ε* is the absorption coefficient; *C* is the concentration; *d* is the averaged path length; *S* is the scattering term.

If it is assumed that no scattering changes in brain tissue occur during activation of the brain, the change in absorption across the activation can be expressed by the following expression:

(2) $$\Delta \text{Abs}=-\log\;\left(\frac{\Delta I_{\text{out}}}{\Delta I_{\text{in}}}\right)=\varepsilon d\Delta C\left(\Delta \text{oxyHb},\Delta \text{deoxyHb}\right).$$

Furthermore, if it is assumed that the change in concentration (Δ*C*) is proportional to the changes in oxygenated hemoglobin (ΔoxyHb) and deoxygenated hemoglobin (ΔdeoxyHb), the following relational expression can be obtained:

(3) $$\Delta \text{Abs}\left(\lambda_{i}\right)=d\left[\varepsilon_{\text{oxy}}\left(\lambda_{i}\right)\Delta \text{oxyHb}+\varepsilon_{\text{deoxy}}\left(\lambda_{i}\right)\Delta \text{deoxyHb}\right].$$

The absorption coefficients of oxygenated hemoglobin and deoxygenated hemoglobin at each wavelength, *ε*_oxy_(*λ*_*i*_) and *ε*_deoxy_(*λ*_*i*_), are known; therefore, *d*ΔoxyHb and *d*ΔdeoxyHb can be obtained by performing measurements with near-infrared rays of two different wavelengths and solving simultaneous equations (3). However, the physical quantity obtained here is the product of the change in concentration and the averaged path length; so care should be taken. 

In general, the averaged path length *d* varies largely from one individual to another and from one part to another. Therefore, caution must be exercised in evaluating the results. 

## 3. Signal Processing Methods for fNIRS

### 3.1. Recording of fNIRS Signal

Mental calculation tasks, low-level task: simple one-digit addition (e.g., 3 + 5), high-level task: subtraction and division with decimal fraction (e.g., 234/(0.61 − 0.35)), were set to obtain fNIRS signal. The brain activity in the prefrontal lobe was measured using fNIRS. The measuring instrument was the multichannel fNIRS instrument, OMM-3000, Shimadzu Corporation, Japan [12].

**Figure 1:**
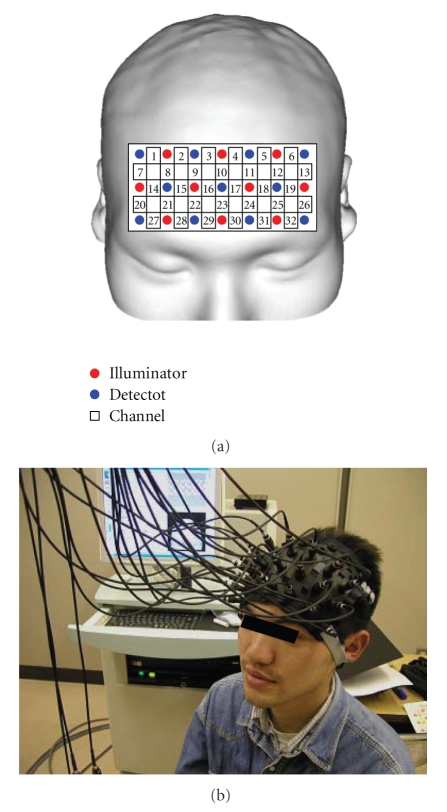
Position of optical fibers and channels in recoding fNIRS signal (Mental calculation task: 3 × 7 matrix, 32 channels).

**Figure 2:**
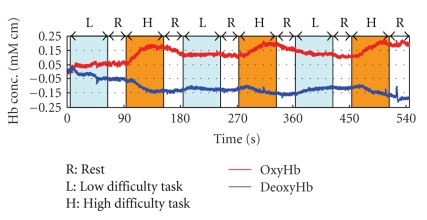
Time history of fNIRS signal in mental calculation (channel number 20).

Figure 1  illustrates the arrangement of optical-fiber units and the location of each channel (3 × 7 matrix, 32 channels). Figure 2  shows the recorded time history of oxygenated hemoglobin (red line, indicated as oxy-Hb) and deoxygenated hemoglobin (blue line, indicated as deoxy-Hb) of channel number 20.

### 3.2. Analysis of fNIRS Signals

In fNIRS analysis, it is necessary to separate noise that is related to a task from that which is not, since fNIRS measures not only the signals of brain activity during a task but also other signals, including measurement noise. 

In general, changes in oxygenated hemoglobin and deoxygenated hemoglobin when the brain is activated and restored to the original state exhibit the trend illustrated in Figure 1  [13]. Therefore, if these signals can be extracted from the measured signals, it is obvious that the brain has been activated. 

Averaging and base-line correction are conventional signal processing methods. These methods require block design, an experimental technique that involves repeating the same stimuli (tasks) and resting multiple times in order to detect brain activation during a task. 

Averaging is the method by which data is averaged for each task. Randomly generated noises approach zero by averaging, and only periodical data is left. Averaging is effective when similar reactions are generated repeatedly. However, for cerebral blood flow that has large scattering of reactions to the same stimuli, the reliability of averaged signals is low, and false signals may be created. Furthermore, it is possible that even significant signals may become undetectable after averaging. 

Base-line correction corrects the start point and end point of a block to zero to remove gentle trends, based on the assumption that blood flow is restored to its original state during a task block. However, because blood flow involves irregular fluctuations, the reference points are unstable. Therefore, if the whole block is corrected based on those two points alone, signals may be distorted. 

Figure 4  shows the result of base-line correction applied for fNIRS signal (Figure 2) after removing high-frequency noise by moving average of 25 data. Figure 5  indicates the functional brain imaging of frontal. It should be noted that the brain activation gradually declines when one subject repeats the same task multiple times.

### 3.3. Decomposition and Reconstruction of fNIRS Signals Using Wavelet Transform

#### 3.3.1. Wavelet Transform

Fourier analysis, which is frequently used for waveform analysis, transforms information in the time domain into information in the frequency domain through the Fourier transform. However, time information is lost in the course of transform. 

Short-time Fourier transform, or windowed Fourier transform, can be used for time-frequency analysis of signals. However, the detecting capacity varies largely, depending on the setting of the window. 

In contrast, wavelet transform is an efficient method of time-frequency analysis. It adapts the window width in time and frequency so that the window width in frequency becomes smaller when the window width in time is large, or the window width in frequency becomes larger when the window width in time is small. 

Wavelet transform expresses the local shape of the waveform to be analyzed, *S*(*t*), by shifting and dilating the waveform called the mother wavelet, *ψ*(*t*), and then analyzes the waveform.

Continuous wavelet transform is given by

(4) $$\left(W_{\psi }S\right)\left(a,b\right)=\int_{-\infty }^{\infty }\frac{1}{\sqrt{\left|a\right|}}\bar{\psi \left(\frac{t-b}{a}\right)}S\left(t\right)dt.$$

In the continuous wavelet transform, information is duplicated, requiring many calculations. Therefore, the method that is expressed by (5), where *a* and *b* are discretized, is called discrete wavelet transform:

(5) $$D_{m}=\int_{-\infty }^{\infty }S\left(t\right)\psi_{m,n}\left(t\right)dt,$$

where

(6) $$\psi_{m,n}\left(t\right)=2^{-m/2}\psi \left(2^{-m}t-n\right).$$

Discrete wavelet transform handles a smaller volume of information than continuous wavelet transform does, but it is able to transform signals more efficiently. Furthermore, its use of an orthonormal base facilitates complete reconstruction of original signals without redundancy. The following section describes decomposition and reconstruction of signals using multiresolution analysis (MRA). 

#### 3.3.2. Multiresolution Analysis (MRA)

MRA decomposes signals into a tree structure using the discrete wavelet transform. MRA decomposes the object time-series signals, *S*(*t*), into an approximated component (low-frequency component) and multiple detailed components (high-frequency components).

Signal *S*(*t*) can be expressed as follows by discrete wavelet transform using an orthonormal base:

(7) $$S\left(t\right)=\overset{\infty }{\underset{n=-\infty }{\sum }}A_{m_{0},n}\phi_{m_{0},n}\left(t\right)+\overset{m_{0}}{\underset{m=-\infty }{\sum }}\;\overset{\infty }{\underset{n=-\infty }{\sum }}D_{m,n}\psi_{m,n}\left(t\right).$$

Here, *ϕ*_*m*,*n*_(*t*) is the scaling function as defined by the following equation.

The coefficient of the approximated component is calculated by

(8) $$A_{m,n}=\int_{-\infty }^{\infty }S\left(t\right)\phi_{m,n}\left(t\right).$$

The detailed components of the signals on level *m* can be expressed by

(9) $$d_{m}=\overset{\infty }{\underset{n=-\infty }{\sum }}D_{m,n}\psi_{m,n}\left(t\right).$$

Thus, the original signal, *S*(*t*), can be expressed as

(10) $$S\left(t\right)=a_{m_{0}}+\overset{m_{0}}{\underset{m=-\infty }{\sum }}d_{m}.$$

Therefore, it is possible to reconstruct task-related components from multiple detailed components.

In the wavelet transform, the choice of a mother wavelet *ψ*_*m*,*n*_(*t*) is important. We employed *Daubechies* wavelet [14], which is orthonormal base and compactly supported wavelet. The vanishing moments of *Daubechies* wavelet can be changed by index *N*. We decided to use a relatively high-order generating index, *N* = 7.

Figure 5  presents the MRA results for oxygenated hemoglobin in channel number 20, where task-related changes were remarkable. Here the measured signal is decomposed into ten levels. The trend of the whole experiment was extracted on the approximated component (*a*_10_), lowest-frequency range. Here, *d*_1_ and *d*_2_, highest-frequency range, had a relatively large amplitude. It is possible that these were measurement noises. Because the interval of repetition of tasks and rests was 64 seconds, the *d*_8_ component was the central component of task-related changes. Therefore, signals were reconstructed by adding the *d*_7_, *d*_8_, and *d*_9_ components. 

**Figure 3:**
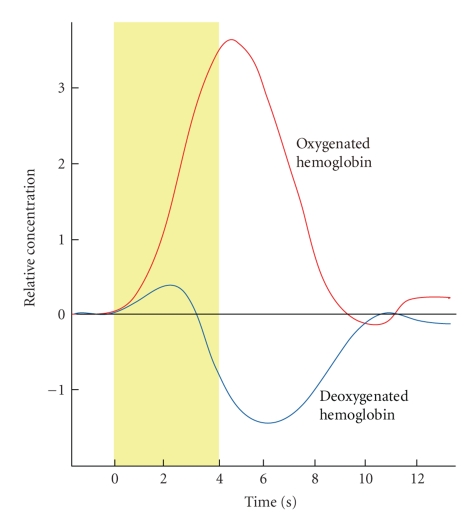
Schematic hemoglobin concentration change due to neural activity.

**Figure 4:**
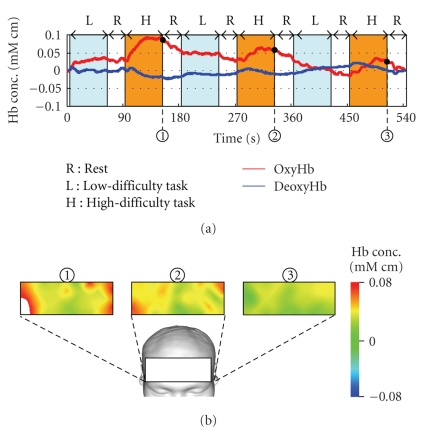
Results of signal processing with base-line correction and denoising.

**Figure 5:**
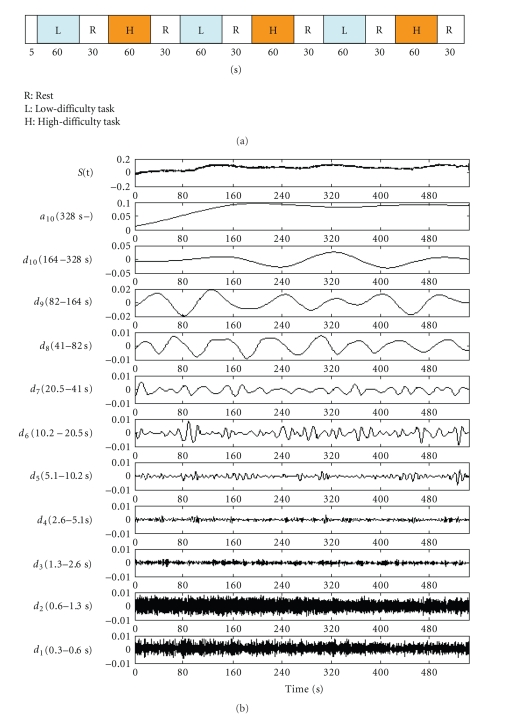
Decomposition of fNIRS signal using MRA.

**Figure 6:**
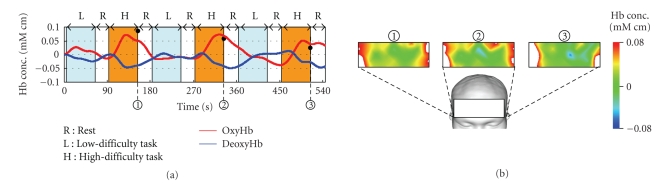
Results of signal processing using MRA.

Reconstructed signals are illustrated in Figure 6. It should be noted that the activation pattern of oxygenated hemoglobin and deoxygenated hemoglobin, shown in Figure 3, is observed very clearly. Comparison between Figure 4  (conventional method) and Figure 6  (proposed method) shows the better performance of the proposed method. Results revealed that oxygenated hemoglobin increased, and the brain was activated during mental calculation tasks. 

## 4. Measurement of Brain Functions under Workload Using Mental Calculation Tasks

To confirm the validity of the signal processing method explained in the previous section, we measured brain functions through simultaneous use of fNIRS and fMRI.

### 4.1. Setting of Workload

To measure brain activity under workload, we used the workload of mental calculation. Mental calculation tasks were set to low, medium, and high levels as follows:

- Low-level task: simple one-digit addition (e.g., 3 + 5);
- Medium-level task: one-digit addition of three numbers (e.g., 6 + 5 + 9);
- High-level task: subtraction and division with decimal fraction (e.g., 234/(0.61 − 0.35)).

**Figure 7:**
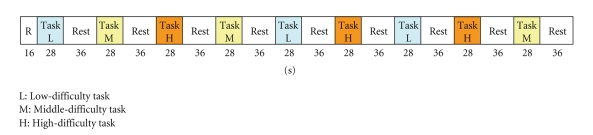
Design of experiment.

The design of the experiment is presented in Figure 7. Each set was composed of 28 seconds of task and 36 seconds of rest in that order. By arranging three sets for each level in random order, a total of nine sets of experiment were conducted over 592 seconds. 

A 28 seconds-task consisted of 14 questions at 2 seconds-intervals for the low level, 10 questions at 2.8 seconds-intervals for the intermediate level, or two questions at 14 seconds-intervals for the high level. The subject answered the questions displayed on the PC screen without speaking. During the 36 seconds-rest time, the subject rested while steadily gazing at the cross mark displayed on the PC screen. 

### 4.2. fNIRS and fMRI Recording

The brain activity in the prefrontal lobe was measured using fNIRS and fMRI simultaneously. fNIRS data were collected on OMM-3000, Shimadzu Corporation, Japan, in MRI scanner.

fMRI data (3 mm thickness, 40 slices) were collected on Siemens Symphony 1.5 T (T2*-weighted gradient-echo sequence, TR = 4000 milliseconds, TE = 50 milliseconds, FA = 90 deg, 64 × 64 pixel, FOV = 192 mm). Whole brain image is obtained as T1-weighted image (TR = 2200 milliseconds, TE = 3.93 milliseconds, FA = 15 deg, TI = 1100 milliseconds, 1 mm^3^ voxel , FOV = 256 mm). 

fMRI data were preprocessed using Statistical Parametric Mapping (SPM99, Welcome Department of Imaging Neuroscience, UK) Normalized contrast images were smoothed with an isotropic Gaussian kernel (FWHM = 12 mm). Regions of interests (ROIs) were defined as clusters of 10 or more voxels in which parameter estimate values differed significantly from zero (*P* < .01).

The subjects were nine healthy men and women. The arrangement of optical fiber units and measurement positions is shown in Figure 1. 

### 4.3. Decomposition and Reconstruction of fNIRS Signals

**Figure 8:**
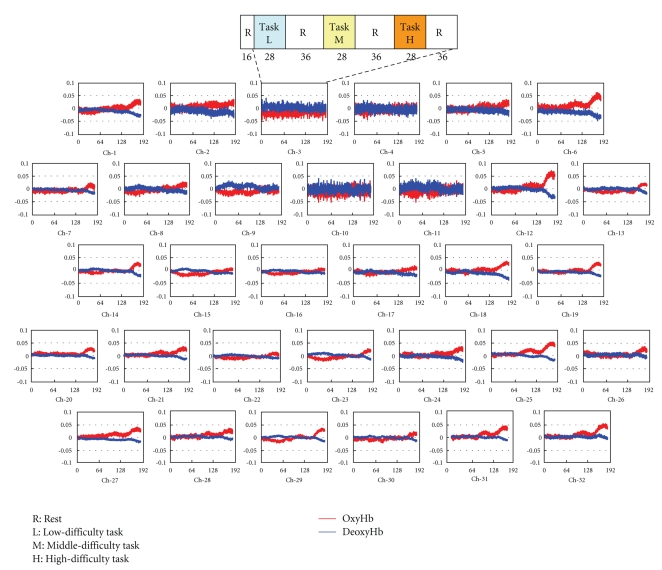
Hemoglobin concentration changes in frontal.

Figure 8  presents the measurement results through all channels for Subject A during the first three tasks. During the mental calculation task at the high level (i.e., the third task), oxygenated hemoglobin increased deoxygenated hemoglobin decreased on both outer sides of the frontal lobe. 

**Figure 9:**
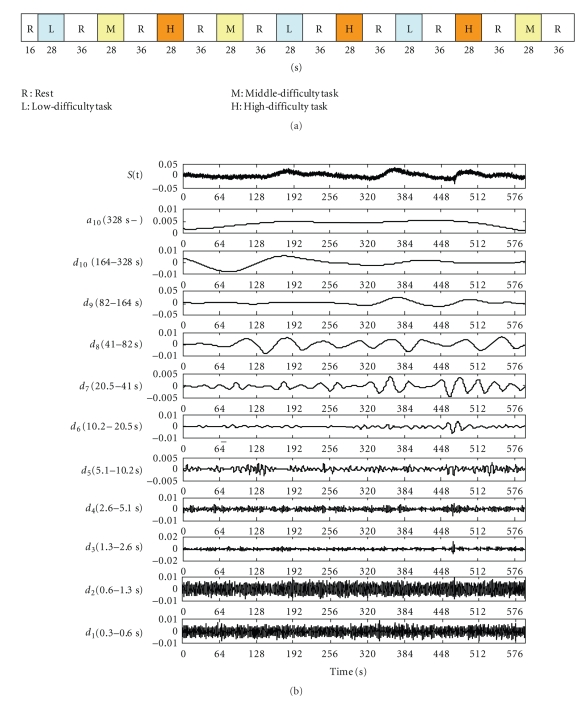
Decomposition of fNIRS signal using MRA (channel number 26).

Figure 9  presents the MRA results for oxygenated hemoglobin in channel number 26, where task-related changes were remarkable. The trend of the whole experiment was extracted on the approximated component (*a*_10_). Because the interval of repetition of tasks and rests was 64 seconds, the *d*_8_ component was the central component of task-related changes. Therefore, signals were reconstructed by adding the *d*_7_, *d*_8_, and *d*_9_ components*．*

**Figure 10:**
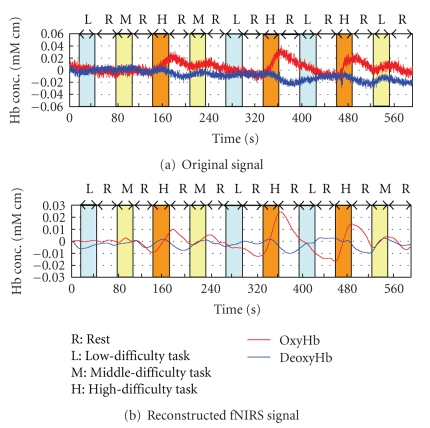
Comparison of original signal and task-related signal (channel number 26).

Reconstructed signals of channel number 26 are illustrated in Figure 10. Results revealed that oxygenated hemoglobin increased, and the brain was activated during mental calculation tasks. Furthermore, such changes became larger as the level of mental calculation task became higher.

**Figure 11:**
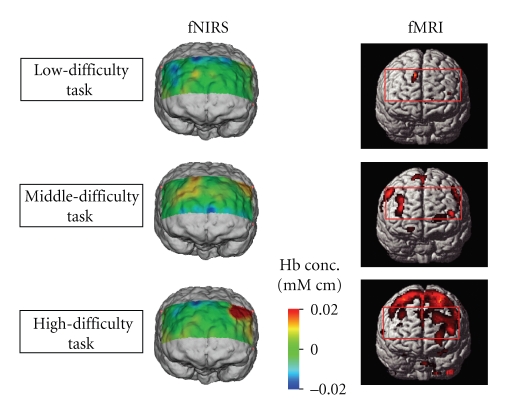
Functional brain imaging by fMRI and fNIRS.

Figure 11  shows the comparison of the functional brain imaging by fMRI and fNIRS with proposed method. The rectangular in fMRI image indicate the region of measurement with fNIRS. The fNIRS images agree to that of fMRI in different workload levels. This results support the effectiveness of MRA with discrete wavelet transform.

### 4.4. Statistical Analysis

The fNIRS signal expresses the quantity of relative changes using the start point as the reference; however, comparisons of measurements between subjects or statistical processing of measurements of all subjects cannot be implemented using this signal as it is. Therefore, we propose a method for converting data of oxygenated hemoglobin and deoxygenated hemoglobin reconstructed by MRA into Z-scores using the following expression, so that the mean value is 0 and standard deviation is 1:

(11) $$Z=\frac{X-\mu }{\sigma }.$$

Here, *X* is the signal of oxygenated hemoglobin or deoxygenated hemoglobin reconstructed using MRA; *μ* is their mean value; *σ* is the standard deviation. 

**Figure 12:**
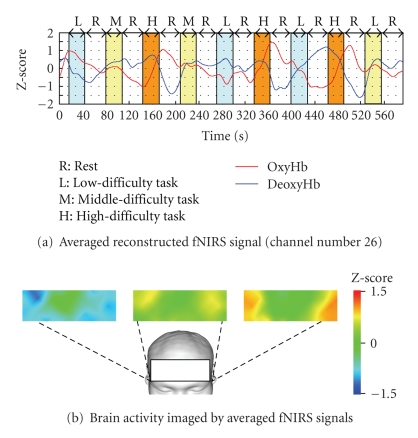
Results of group analysis of fNIRS signals for nine subjects.

**Figure 13:**
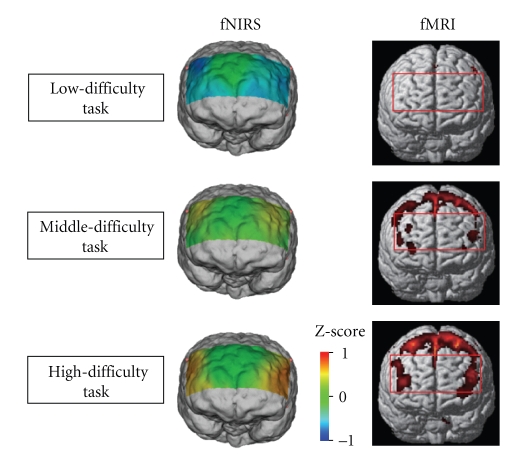
Functional brain imaging by fMRI and fNIRS (Group analysis for nine subjects).

Figure 12  shows the averaged fNIRS signals using Z-score for nine subjects. It should be noted that the difference of the workload level is reflected on the gradient of oxygenated hemoglobin concentration. Figure 13  shows the results of group analysis for nine subjects. The rectangular in fMRI image indicates the region of measurement with fNIRS. The fNIRS images agree to that of fMRI in different workload levels. This results support the effectiveness of the proposed method.

### 4.5. Subjective and Objective Evaluation of Workload

In this experiment, the workload of each subject was measured using the Japanese version of NASA-TLX to evaluate the correlations between the workload of mental calculation tasks and the objective evaluation with fNIRS. NASA-TLX is composed of six measures: mental requirements, physical requirements, temporal demand, work performance, effort, and frustration. Before workload evaluation, the subject performed one-to-one comparisons of the importance of elements of the workload involved in task performance. 

The weight of each measure was based on the number of times an element was selected as more important during 15 one-to-one comparisons. When evaluating the workload of each task, the subject placed a mark at the appropriate position on the segment drawn between both extremes for each of the six measures. 

A Weighted Workload (WWL) score was obtained by reading the position of each evaluation mark on a scale of 0 to 100 and multiplying it by the weight for each measure determined by one-to-one comparison, and then averaging all the products. 

**Figure 14:**
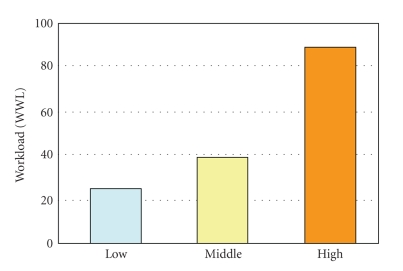
Workload evaluation by NASA-TLX.

Figure 14  presents the WWL score of Subject A as determined by NASA-TLX. The workload became higher when the task level was higher. 

**Figure 15:**
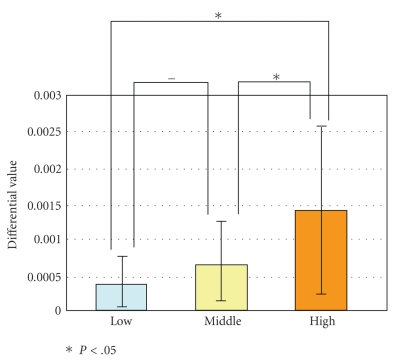
Maximum gradient of fNIRS signal during the task.

Figure 15  shows the results of 9 subject evaluated using the maximum gradient of oxygenated hemoglobin in the task with different workload level. Multivariate test using Ryan method is used. Significant difference between high-level task and low-level task or between high-level task and medium-level task can be observed (*P* < .05). It exhibited good correlation with subjective evaluation with NASA-TLX. This result confirmed the feasibility of evaluating workload using the signal of cerebral blood flow obtained from fNIRS. 

## 5. Measurement of Brain Functions of Car Driver

Drivers of motor vehicles obtain visual information on the surrounding environment, recognize and judge that information suitably, and then control their vehicle through steering wheel, accelerator, and brake pedal operations. Human brain activity functions to control all of these processes. In situations where it is necessary to predict unexpected danger, it is thought that a driver's brain activity strengthens the cognition function by spontaneously raising the level of attention. In the course of developing driver support systems, it is important to have a clear understanding of human brain activity in such driving situations. 

### 5.1. Contents of the Task

**Figure 16:**
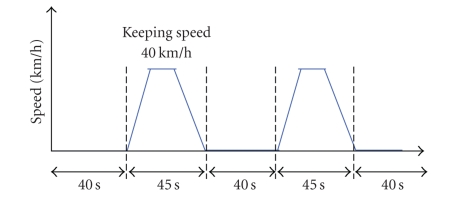
Speed pattern of leading car.

To verify that the driving workload reduction of Adaptive Cruise Control (ACC) could be evaluated from brain activity, we conducted an experiment that involved the use of a driving simulator to follow a vehicle (Figure 16). 

Main specification of the driving simulator is as follows: Dimension: 2440 mm (*W*)∗ 2280 mm (*H*)∗ 1850 mm (*D*), front view: wide filed (138 degrees) screen projection, DLP projector with total pixel count of 780000 (XGA), rear view: 3 mirror independent LCD display 640 ∗ 480 pixel (VGA), computer graphics: redraw speed: 30 to 60 flame/s, and simulation system: 6 axis motion base system using 6 electric screw cylinders.

Driving tests were conducted under two conditions: one involved following a vehicle by utilizing ACC, and the other involved following a vehicle while driving without ACC. The subject performed practice runs to become somewhat skillful in handling the driving simulator and then drove two times under each condition. Brain activity during one condition was compared with that during the other condition. 

### 5.2. Measurement Method

**Figure 17:**
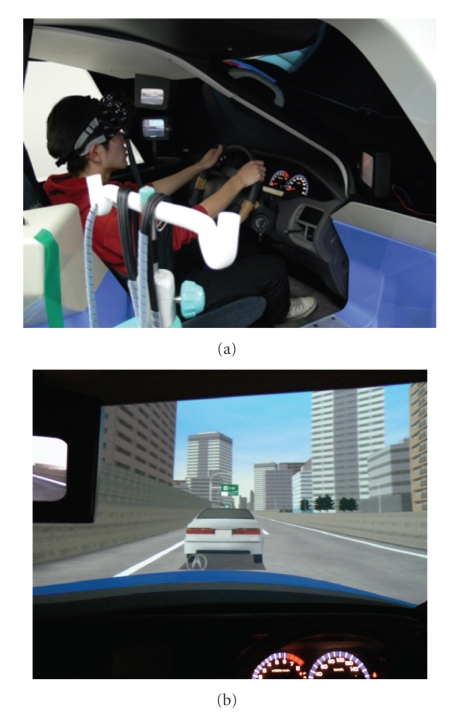
Experiment with driving simulator (driver follows the proceeding vehicle with and without ACC).

**Figure 18:**
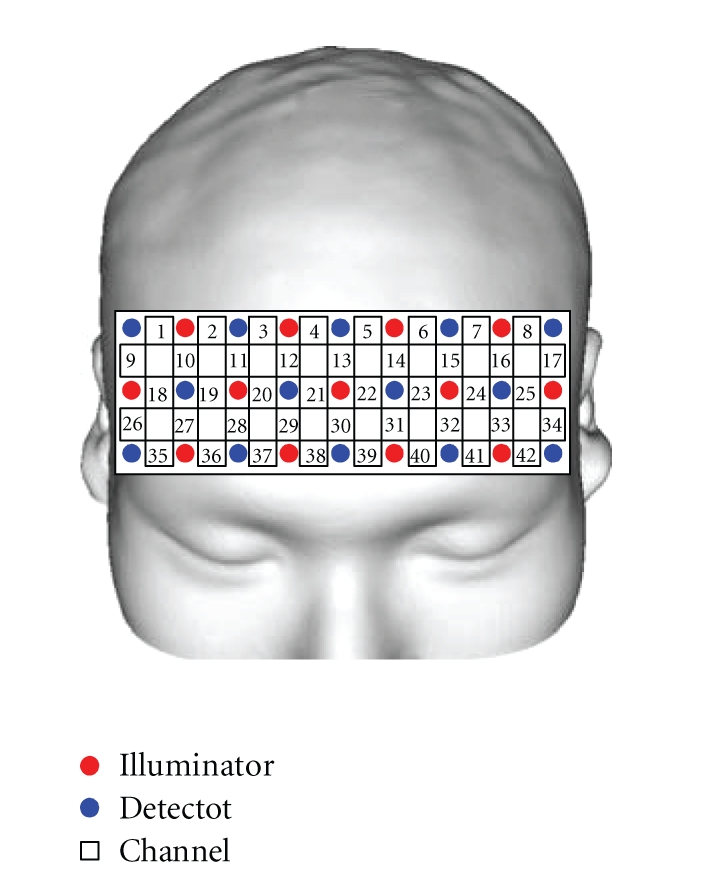
Position of optical fibers and channels (driving task: 3 × 9 matrix, 42 channels).

Brain activity in the frontal lobe was measured using fNIRS. Figure 17  depicts a scene of the experiment. The measuring instrument was a near-infrared imaging device, OMM-300, Shimadzu Corporation, Japan. Figure 18  illustrates the arrangement of optical-fiber units (3 × 9 matrix, 42 channels). The numbers between the light-emitting fiber unit and the light-receiving fiber unit denote the measurement channels; measurements were performed through a total 42 channels. Furthermore, driving performance was also recorded on the driving simulator while measuring brain activity. The four male subjects, who were in their 20 s in healthy condition and who had ordinary driving licenses, participated. 

### 5.3. Decomposition and Reconstruction of fNIRS Signals

The fNIRS signals include signals that are not related to brain activity (e.g., noise of the measurement instrument, influences of breathing, and changes in blood pressure). It was necessary to remove these unrelated signals to evaluate brain activity in detail. Therefore, the measured fNIRS signals were decomposed through MRA using discrete wavelet transform, and the components related to the driving task were reconstructed. Then, group analysis using Z-score was conducted for all subjects.

### 5.4. Results

**Figure 19:**
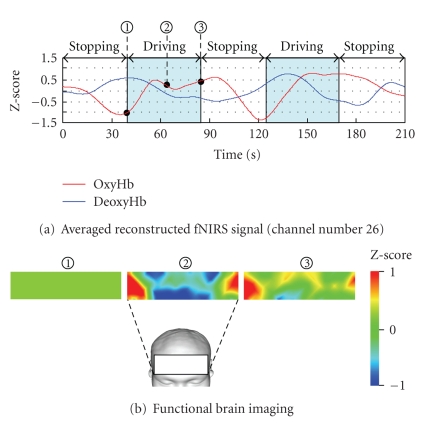
Result of group analysis for four drivers without ACC system.

**Figure 20:**
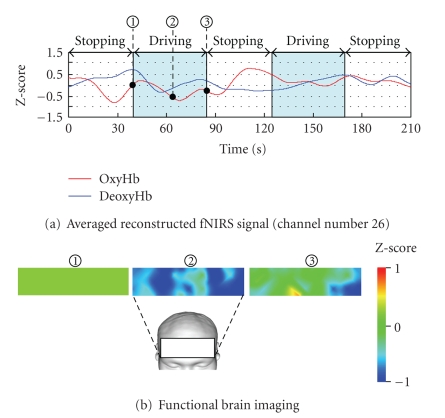
Result of group analysis for four drivers with ACC system.

Figures 19  and 20  depict the relationships between brain activity when the subject followed the forerunning vehicle manually without using ACC and that when the subject used ACC (26 channels at the outer right portion of the frontal lobe) and vehicle speed.

Figure 19  presents the result of group analysis for four drivers without ACC, and Figure 20  presents the result with ACC. Figure 19(a)  confirms that oxygenated hemoglobin increased when the subject drove without ACC and exhibited a high value in the latter half of the task. The brain function imaged in Figure 19(b)  confirms that, as common brain activity, both outer portions of the frontal lobe became active during the driving task. 

Figure 20(a)  indicates that oxygenated hemoglobin did not increase while driving with the use of ACC. Also, the brain function image in Figure 20(b)  reveals that the frontal lobe was less active than when the subject drove without ACC. This result may reflect the reduction of driving workload by ACC.

## 6. Conclusions

Signal processing method to extract the task-related components with multiresolution analysis (MRA) based on discrete wavelet transform is proposed for fNIRS. Then the integration of data of multiple subjects using Z-scores is developed for statistical group analysis.

The brain activity of the subject who was given workload by different levels of mental calculation tasks was measured with fNIRS and fMRI. The fNIRS images constructed with the proposed method agree to fMRI images in different workload levels. Those results show that the proposed method is effective for evaluation brain activity measured by fNIRS.

The changes in brain activity in connection with workload were compared with the subjective evaluation of workload by NASA-TLX. Good correlation was observed between the brain activity detected by fNIRS and the workload scores obtained from NASA-TLX. This result indicates that it is possible to evaluate workload from the cerebral blood flow signals obtained from fNIRS. 

Whether the reduction of driving workload by ACC can be evaluated from brain activity was evaluated through experiments using a driving simulator. The results revealed that while the outer portions of the frontal lobe were active in connection with driving performance when the subject drove without ACC, it indicated no activity related to driving performance with the use of ACC. These results suggest the possibility of evaluating driving assistance systems through evaluation of the driving workload from measurement of brain activity using fNIRS. 

Neuroimaging studies of car drivers using fNIRS should be conducted with increased number of subjects. We cannot conclude that lowering brain activity by reducing driving workload leads to safe driving; thus, in the future, we will design and evaluate driving assistance systems that require an appropriate level of brain activity.
